# Residual Performance and Biomechanical Asymmetries During Jumping Tasks in Female Athletes at 9 Months After Anterior Cruciate Ligament Reconstruction

**DOI:** 10.1177/23259671241276826

**Published:** 2024-10-09

**Authors:** Berit Flora Warnecke, Chris Richter, Enda King, Florian Kurt Paternoster

**Affiliations:** † Technical University of Munich, Munich, Germany; ‡ Sports Surgery Clinic, Dublin, Ireland; § Aspetar, Doha, Qatar; Investigation performed at Sports Surgery Clinic, Dublin, Ireland

**Keywords:** anterior cruciate ligament, return to sport, athletic performance, biomechanics, female

## Abstract

**Background:**

Biomechanics and anterior cruciate ligament injury mechanisms differ in males and females. There is a need for more data on between-limb biomechanical differences after anterior cruciate ligament reconstruction (ACLR) in females.

**Purpose:**

To explore biomechanical asymmetries throughout the kinetic chain during the single-legged (SL) and double-legged (DL) countermovement jump (CMJ) and drop jump (DJ) in female athletes after ACLR.

**Study Design:**

Descriptive laboratory study.

**Methods:**

Kinematic and kinetic between-limb differences were analyzed during the SL and DL CMJ and DJ in 67 female athletes 9 months after ACLR. Biomechanical and performance asymmetries between limbs during the jumps and isokinetic strength testing were analyzed with statistical parametric mapping. The entire stance phase was used for the paired *t* tests of the biomechanical variables, with Cohen *d* effect sizes of significant portions of the stance phase (reported as % of stance) calculated in a point-by-point manner.

**Results:**

Decreased vertical ground-reaction force, internal knee abduction moment, knee internal rotation angle, hip external rotation angle, internal ankle eversion, and external rotation moments were seen in the ACLR limb during all 4 vertical jump tests. The greatest number and highest value of differences were found during the DLDJ, with asymmetries having medium to large effect sizes. They tended to appear more frequently in the concentric phase (50% to 100% of stance) during the SLCMJ and DLCMJ and in the eccentric (0% to 49% of stance) and concentric (50% to 100% of stance) phase during the SLDJ and DLDJ. For the SLCMJ, SLDJ, and quadriceps strength, performance asymmetries of >15% were detected but not for change of direction.

**Conclusion:**

The findings suggest that return-to-play testing in female athletes should examine the entire stance phase and include assessments of kinetic and kinematic variables throughout the kinetic chain. Greater deficits were highlighted in the DJ than in the CMJ, and greater performance asymmetries were evident in the SL tasks, with greater kinetic and kinematic and compensatory strategies evident in the DL tests.

**Clinical Relevance:**

Biomechanical analysis focusing on contralateral compensation strategies and sex-specific interventions are necessary before return to play.

Anterior cruciate ligament (ACL) rupture is a devastating injury associated with short-term functional constraints and long-term morbidity.^26,38^ Such ruptures are frequent in pivoting and landing sports, with a 4 to 6 times higher incidence in females than males.^10,12^ After a rupture, ACL reconstruction (ACLR) is standard for young athletes aiming to return to multidirectional sport.^23^ Incomplete rehabilitation with residual physical deficits after ACLR can influence outcomes of return to play, knee pain, and second ACL injury.^6,14^

Decision-making for return to play after ACLR is multifactorial and challenging.^39^ Common testing procedures focus on assessing lower limb strength and functional performance symmetry among limbs, such as measuring jump height. In addition, temporal rehabilitation guidelines,^21^ with a common return-to-play target of 9 months postoperatively,^14,18^ are frequently employed. However, it is worth noting that validated return-to-play tests, which evaluate rehabilitation progress, exhibit superior effectiveness in younger males when compared with females.^36^ Finding normal limb asymmetry for performance (jumps <15%, strength <10%) across a test battery is associated with a lower risk of ACL reinjury.^9,22^ Muscle strength asymmetries are commonly tested using isokinetic devices,^8,14^ whereas for jumping performance, the single-legged (SL) and double-legged (DL) countermovement jump (CMJ) and drop jump (DJ) are often used.^4,7,18^ The DLDJ has been found to be a reliable and valid instrument in observing dynamic knee abduction,^7^ which plays an important role in rehabilitation.^11,12,20,31^ However, performance symmetry rehabilitates faster in change of direction (CoD) than vertical-jump testing.^19^ Despite the recovery of CoD and jump performance, ongoing biomechanical asymmetries can persist between limbs after ACLR^13,37^ and should thus be included as a criterion for return to play.^6,8,13,21,26^

Numerous studies have demonstrated biomechanical between-limb differences at the return-to-play time point in males.^13,14,16,18^ Differences were observed in knee frontal and transverse plane kinetics, ankle transverse plane kinetics, and kinematics during the DLDJ as well as in knee frontal plane kinetics and the posterior center of mass (CoM) position during the SLDJ.^16^ In comparison, few studies have examined biomechanical between-limb differences after ACLR in females. Paterno et al^29^ reported decreased peak vertical ground-reaction force (GRF) in the ACLR limb compared with the non-ACLR limb in the drop vertical jump in females. Furthermore, during SL horizontal jumping, a decrease in peak knee flexion, hip flexion, and hip extension moment was observed for both sexes when comparing the ACLR limb with the non-ACLR limb^37^ and with a healthy control group.^34^ A limitation of many studies in the area is the focus on discrete values, which can neglect relevant differences and further information that may be apparent during analysis of the entire stance phase.^28,32^ Furthermore, peak values can occur at varying points in the stance phase, making comparison prone to error.

Biomechanical differences between the sexes are known in the healthy population. Females have reduced hip control compared with males, which alters lower limb biomechanics.^25^ However, studies report inconsistent results for sex-based differences in the knee and hip sagittal planes as well as for vertical and posterior GRF.^20,25,29^ Moreover, ACL injury mechanisms differ between males and females. Krosshaug et al^20^ described a 5.3-times higher relative risk of sustaining a knee abduction collapse for female basketball players compared with their male counterparts. A collapsing knee abduction is a common noncontact injury mechanism, which may explain the increased incidence in females than in males.^11,12,20,31^ Shimokochi and Shultz^33^ found that knee external rotation moment combined with quadriceps force increases ACL strain, especially near full knee extension. Furthermore, ACL strain was found to be higher when a knee abduction load was combined with knee internal rotation.^20,33^ However, the combination of external rotation and knee abduction may lead to ACL impingement, another injury mechanism^20,33^ detected predominately in females.^4,20^

The purpose of the current study was to identify biomechanical asymmetries throughout the kinetic chain during SL and DL CMJ and DJ in female athletes after ACLR, so as to inform more targeted rehabilitation. We hypothesized that between-limb differences would exist in all exercises and planes, especially in the frontal plane of the knee, but with different occurrences within the stance phase. In addition, we hypothesized that performance asymmetries would be lower during CoD testing compared with CMJ, DJ, and strength testing.

## Methods

### Study Participants

Study participants were recruited before undergoing ACLR from July 2014 to September 2018 as part of a long-term follow-up study. The inclusion criteria were (1) females aged 18 to 35 years with 8 to 10 months of follow-up, (2) athletes from any level of multidirectional sports who preoperatively self-declared their expectation to return to the same level of play or higher, and (3) participants who underwent ACLR with bone–patellar tendon–bone and hamstring (semitendinosus/gracilis) tendon autografts from the ipsilateral side. Exclusion criteria were meniscal or additional ligament repair during surgery and previous ACL injury. The patients underwent ACLR by 1 of 3 orthopaedic consultants specializing in knee surgery at our institution. Local clinicians and physiotherapists guided individual rehabilitation. This study was performed according to the Declaration of Helsinki, the study protocol received ethics committee approval, and written informed consent was obtained from all participants. The study was registered at ClinicalTrials.gov (NCT02771548).

### Physical Testing Setup

The participants underwent vertical jump tests (DLCMJ, SLCMJ, DLDJ, and SLDJ), planned and unplanned 90° CoD tests, and then strength tests. All testing was performed at the 3-dimensional biomechanics laboratory of our institution; participants wore their own athletic footwear during testing. An 8-camera motion analysis system (200 Hz; Bonita-B10, Vicon) including 2 force platforms (1000 Hz; BP400600, AMTI) was used to analyze the jump tests. Data were synchronously recorded using Vicon Nexus software (Version 1.8.5; Vicon Motion Systems Ltd.). A modified plug-in gait model was used,^24^ without considering the arm and head segments for kinematic and inverse dynamic analysis. The planned and unplanned 90° CoD tests were performed with a start gate placed 2 m from the start line, triggering the exit gates 2 m left and right of the force plates, which were 3 m from the start gate.^15^ The completion time of the CoD tests was measured using speed gates (Smartspeed, Fusion Sport). A motor-driven dynamometer (IKD; Cybex Humac NORM, CSMI) was used to measure the concentric knee extensor (quadriceps) and flexor (hamstrings) torque at an angular velocity of 60 deg/s^4,35^. The range of motion was set to 0° to 100° of knee flexion.

### Physical Testing Protocol

Participants started with a standardized warm-up of a 2-minute submaximal run, determining the pace individually, and 5 unloaded squats. The test protocol included 2 submaximal familiarization trials for every exercise. First, jumps including the DLCMJ, SLCMJ, and DLDJ from a 30-cm step and the SLDJ from a 20-cm step were assessed. Participants were instructed to focus besides jumping as high as possible on short ground contact during the DJ and extended legs during the CMJ. DL jumps were executed with feet hip-width apart, each foot placed on 1 force plate. For consistency, all jumps were executed with the hands on the hips.

Second, the planned and unplanned 90° CoD were performed. For planned CoD, participants were told in advance which exit to use, whereas for unplanned CoD, participants had to react after the start gate triggered the exit gates. Third, concentric knee extensor and flexor strength were measured.

All exercises were explained beforehand and could be declined by the participant or investigator if they believed the test could not be executed correctly or without injury or if the participant did not consent. The non-ACLR limb was always tested first, with a standardized recovery of 30 seconds, until 3 valid trials with complete foot contact on the force plate and maximal effort of the participant were recorded. Staff was trained in cueing and each data set was screened for quality by 2 persons.

### Data Analysis

A low-pass, zero-lag, fourth-order Butterworth filter with a cutoff frequency of 15 Hz^19^ was applied to all raw data. Further calculation of additional kinematic measures and statistical analysis were completed during data processing using custom software (MathWorks). The mean of 3 valid trials was analyzed.

### Data Analysis of Strength and Performance Parameters

In the strength analysis, the variable of interest for concentric knee flexion and extension contractions was peak torque normalized to body mass. In the analysis of CoD, both ground contact time and completion time (all times measured in seconds) were captured. For the jump tests, the analysis integrated measures such as jump height (in meters), ground contact time, and reactive strength index (RSI). Jump height was calculated by the impulse-momentum relationship, with takeoff velocity derived from integrating the acceleration-time signal obtained from the vertical GRF-time signal. In addition, the ground contact time and RSI (measured as jump height/ground contact time) were analyzed for the DJ. The ground contact phase for the DJ was defined as GRF >20 N. Furthermore, the modified RSI (RSI_mod_; measured as jump height/stance phase time) was calculated for the CMJ. The stance phase for the CMJ was defined as the time between the start of the jump (GRF <98% of body weight) and the takeoff (GRF <10 N). For the performance variables of the SL measurements, values of the ACLR limb were divided by the non-ACLR limb and multiplied by 100 to calculate the limb symmetry index (LSI).

### Data Analysis of Biomechanical Differences

Using Vicon Nexus software, internal joint moments were calculated in all 3 planes with standard inverse dynamics procedures. Internal joint moments as well as GRF were normalized to body mass. In addition to analyzing joint kinematics and kinetics during jumps, the relationships between the trunk and pelvis as well as foot angle to the pelvis were calculated in the transverse plane. In addition, the CoM was calculated using the plug-in-gait model.^16^ The stance phase was time-normalized for statistical parametric mapping (SPM). To ensure a correct continuous waveform analysis, the jump data were separated into 2 aligned halves. This segmentation into eccentric and concentric phases facilitates a proper comparison of neuromuscular characteristics across limbs and participants. The eccentric phase is the downward phase and spans from the onset of movement until the vertical velocity reaches zero. Afterward, the concentric or propulsive phase starts, in which the athletes propel their CoM vertically.

### Statistical Analysis

All data were tested for normal distribution and further evaluated if that was not the case. One-dimensional (1D) paired *t* tests for between-limb differences were determined using SPM software (SPM spm1d Version M.0.4.3 [2017.01.28]; http://www.spm1d.org) with 95% CIs; α = 0.05. The entire stance phase was used for the 1D paired *t* tests of the biomechanical variables. Since multiple joints and planes were analyzed, it was essential to identify meaningful differences between limbs and avoid overreporting. Therefore, Cohen *d* effect sizes (ESs) of significant phases were calculated in a point-by-point manner, and only phases of ≥5 frames with each point ES ≥0.5 (medium ES, 0.5-0.69; large ES, ≥0.7) were reported.^2^ King et al^16^ analyzed the complete stance in a similar study but reported all significant phases longer than 5 frames with a mean ES of >0.5. We believed that this leads to less-specific results and might leave critical phases undetected. The start and end within the movement cycle was specified as a percentage of the stance phase. Internal joint moments are stated as moments.

All values are reported relative to the non-ACLR limb and described as mean ± standard deviation, 95% CI, *P* value (specified if <.001), and ES of the phase or discrete point. All results from the performance variables, including LSIs, are reported. For each jump test, biomechanical variables with the strongest ES differences are presented.

## Results

A total of 67 female athletes were included (mean ± SD: age, 22.18 ± 4.23 years; weight, 65.98 ± 6.20 kg; height, 167.15 ± 4.81 cm), at a mean of 9.13 ± 0.76 months after ACLR. All participants had autografts from the ipsilateral side, of which 56 were a bone-patellar tendon-bone graft and 11 a hamstring (semitendinosus/gracilis) tendon graft. Data with valid trials were analyzed with the following sample sizes: n = 67 for isokinetic knee extension and flexion; n = 60 for the DLCMJ; n = 59 for the SLCMJ; n = 64 for the DLDJ; n = 67 for the SLDJ; n = 53 for planned CoD; and n = 49 for unplanned CoD.

### Performance Differences Between Limbs

The results of the performance tests are listed in Table 1. Jump height (LSI = 82.93%) and RSI_mod_ (LSI = 82.22%) during the SLCMJ were decreased significantly in the ACLR limb (*P* < .001 for both). The ACLR limb had significantly less jump height (LSI = 79.27%; *P* < .001), RSI (LSI = 76.31%; *P* < .001), and longer contact time (LSI = 103.50%; *P* = .026) during the SLDJ. Participants showed an LSI close to 100% for planned and unplanned CoD completion time (planned CoD LSI = 100.08%; unplanned CoD LSI = 102.15%) and contact time (planned CoD LSI = 100.50%; unplanned CoD 100.80%). In contrast, the analysis of the isokinetic dynamometry showed that the ACLR limb had significantly less strength during knee extension (quadriceps LSI = 82.53%; *P* < .001) and knee flexion (hamstrings LSI = 92.9%; *P* < .001).

**Table 1:** Differences Between the ACLR and Non-ACLR Limbs During Functional Performance Testing^*a*^

Variable	ACLR		Non-ACLR		LSI, %	*P*	ES
	Mean ± SD	95% CI	Mean ± SD	95% CI			
SLCMJ							
Jump height,^*b*^ cm	7.59 ± 1.95	7.11-8.07	9.15 ± 2.04	8.65-9.65	82.93	**<.001**	−0.73
RSI_mod_, cm/s	0.10 ± 0.04	0.09-0.11	0.12 ± 0.04	0.11-0.13	82.22	**<.001**	−0.54
SLDJ							
Jump height,^*b*^ cm	8.97 ± 2.05	8.47-9.47	11.32 ± 2.40	10.73-11.9	79.27	**<.001**	−0.93
Contact time, s	0.35 ± 0.06	0.34-0.37	0.34 ± 0.06	0.32-0.35	103.50	**.026**	0.19
RSI, cm/s	0.26 ± 0.07	0.25-0.28	0.35 ± 0.09	0.32-0.37	76.31	**<.001**	−0.88
Planned CoD							
Completion time, s	1.50 ± 0.19	1.46-1.55	1.50 ± 0.20	1.45-1.55	100.08	.971	0.01
Contact time, s	0.31 ± 0.04	0.30-0.32	0.31 ± 0.05	0.30-0.32	100.50	.778	0.03
Unplanned CoD							
Completion time, s	1.61 ± 0.12	1.58-1.64	1.57 ± 0.22	1.52-1.63	102.15	.211	0.19
Contact time, s	0.33 ± 0.04	0.33-0.34	0.33 ± 0.05	0.32-0.34	100.80	.681	0.06
Strength,^*c*^ N·m/kg							
Hamstrings^*d*^	1.32 ± 0.23	1.26-1.37	1.42 ± 0.24	1.36-1.48	92.90	**<.001**	−0.42
Quadriceps^*d*^	2.00 ± 0.36	1.91-2.08	2.42 ± 0.33	2.34-2.50	82.53	**<.001**	−1.04

a Boldface *P* values indicate statistically significant difference between the ACLR and non-ACLR limb (*P* < .05). ACLR, anterior cruciate ligament reconstruction; CoD, change of direction; ES, effect size; LSI, limb symmetry index; RSI, reactive strength index; RSI_mod_, modified reactive strength index; SD, standard deviation; SLCMJ, single-legged countermovement jump; SLDJ, single-legged drop jump.

b Calculated with impulse moment.

c Maximal isokinetic strength.

d All values represent body mass-normalized torque.

### Biomechanical Differences Between Limbs

For most biomechanical variables, the non-ACLR limb showed larger values compared with the ACLR limb (Table 2). The greatest number of between-limb differences with large ESs (Table 2, red shading) was found during the DLDJ. All 4 vertical jump tests showed medium or large ESs for vertical GRF, knee abduction moment, knee internal rotation angle, hip external rotation angle, ankle eversion, and external rotation moments (Table 2, yellow shading).

**Table 2:** Summary of Biomechanical Between-Limb Differences During the SL and DL CMJ and DJ

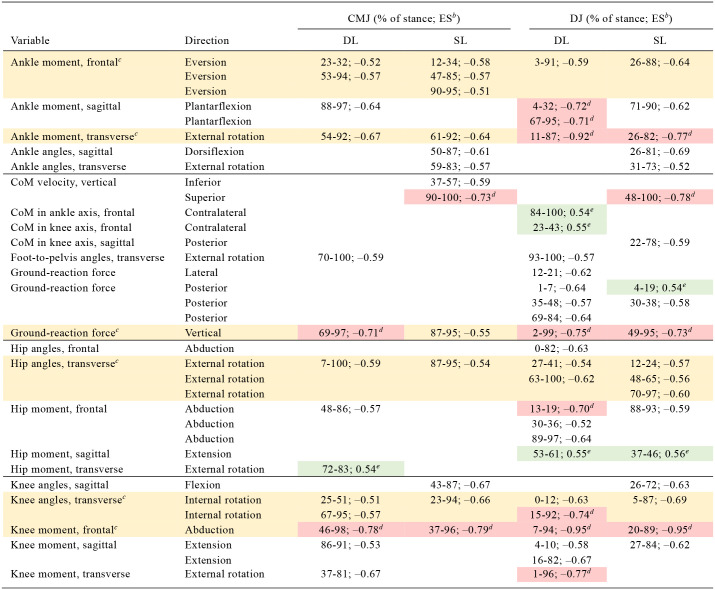

a CMJ, countermovement jump; CoM, center of mass; DL, double-legged; ES, effect size; SL, single-legged.

b *P* < .001 applies for all results.

c Differences for this variable exist in all exercises (yellow shading).

d Significant difference with a large effect size (red shading).

e ACLR > non-ACLR (green shading).

Vertical GRF between-limb differences were found during 69% to 97% of stance for the DLCMJ, 87% to 95% of stance for the SLCMJ, 2% to 99% of stance for the DLDJ, and 49% to 95% of stance for the SLDJ. Knee abduction moment differences were found during 46% to 98% of stance for the DLCMJ, 37% to 96% of stance for the SLCMJ, 7% to 94% of stance for the DLDJ, and 20% to 89% of stance for the SLDJ. Knee internal rotation angle differences were found during 25% to 51% and 67% to 95% of stance for the DLCMJ, 23% to 94% of stance for the SLCMJ, 0% to 12% and 15% to 92% of stance for the DLDJ, as well as 5% to 87% of stance for the SLDJ. Hip external rotation angle differences were found during 7% to 100% of stance for the DLCMJ, 87% to 95% of stance for the SLCMJ, 27% to 41% and 63% to 100% of stance for the DLDJ, as well as 12% to 24%, 48% to 65%, and 70% to 97% of stance for the SLDJ. Ankle eversion moment differences were found during 23% to 32% and 53% to 94% of stance for the DLCMJ, 12% to 34%, 47% to 85%, and 90% to 95% of stance for the SLCMJ, 27% to 41% of stance for the DLDJ as well as 12% to 24% of stance for the SLDJ. External rotation moment differences were found during 54% to 92% of stance for the DLCM, 61% to 92% of stance for the SLCMJ, 11% to 87% of stance for the DLDJ, and 26% to 82% of stance for the SLDJ.

### Double-Legged Countermovement Jump

**Figure 1.**
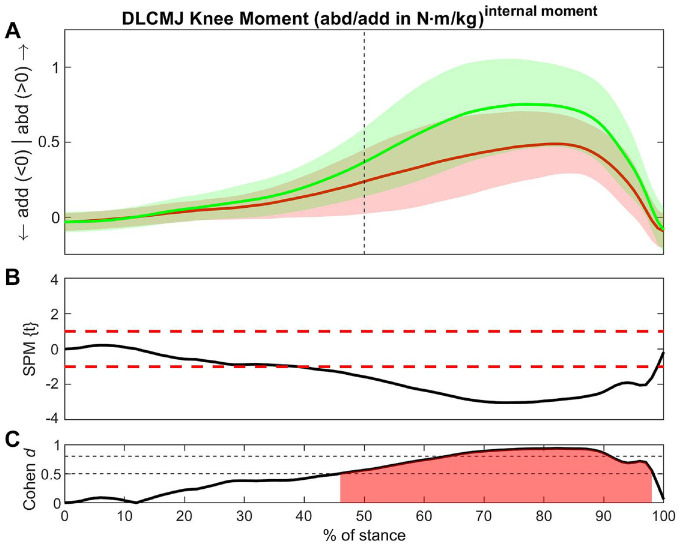
Differences in knee abduction moment between the ACLR and non-ACLR limbs during the double-legged countermovement jump. (A) Mean and SD clouds for the ACLR (red) and non-ACLR (green) limbs as a reference for movement. Knee adduction (negative values) and knee abduction (positive values) are centered around 0, with arrows pointing to the direction of movement. (B) The *t* statistic as a function of time describing the difference between the limbs. The curve exceeding the dashed red lines at >1 and <-1 (representing *P* < .05) indicated that a significant difference exists between the ACLR and non-ACLR limbs. (C) The effect size as a function of time describing the magnitude of the effect. The dotted black lines indicate the percentage of the stance with Cohen *d*≥ 0.5, with areas shaded in red indicating a strong effect size throughout that phase. The between-limb asymmetry was significantly different with a strong effect size from 46% to 98% of the stance in the later part of the eccentric phase until shortly before takeoff. abd, knee abduction; ACLR, anterior cruciate ligament reconstruction; add, knee adduction; DLCMJ, double-legged countermovement jump; SPM, statistical parametric mapping.

The largest ESs were found in knee abduction moment (46-98% of stance; ES = −0.78) (Figure 1) and vertical GRF (69-97% of stance; ES = −0.71) (Table 2, red shading). Compared with the non-ACLR limb, the ACLR limb had larger values in the external hip rotation moment (72-83% of stance; ES = 0.54) (Table 2, green shading).

### Single-Legged Countermovement Jump

**Figure 2.**
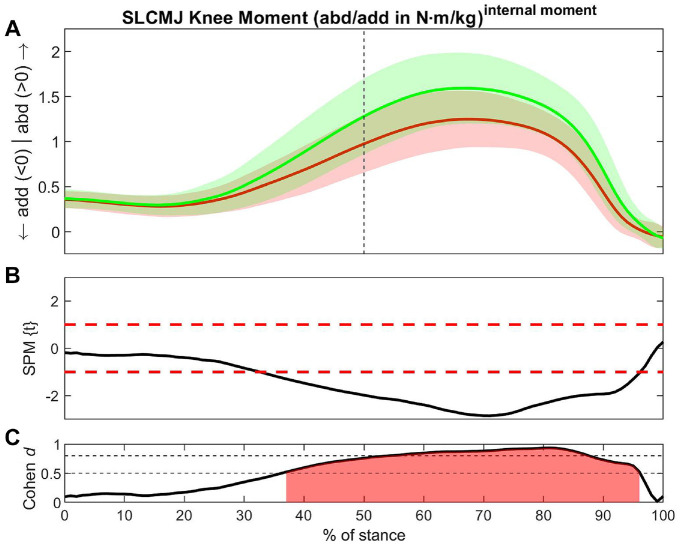
Differences in knee abduction moment between the ACLR and non-ACLR limbs during the single-legged countermovement jump. (A) Mean and SD clouds for the ACLR (red) and non-ACLR (green) limbs as a reference for movement. Knee adduction (negative values) and knee abduction (positive values) are centered around 0, with arrows pointing to the direction of movement. (B) The *t* statistic as a function of time describing the difference between the limbs. The curve exceeding the dashed red lines at >1 and <-1 (representing *P* < .05) indicated that a significant difference exists between the ACLR and non-ACLR limbs. (C) The effect size as a function of time describing the magnitude of the effect. The dotted black lines indicate the percentage of the stance with Cohen *d*≥ 0.5, with areas shaded in red indicating a strong effect size throughout that phase. The between-limb asymmetry was significantly different with a strong effect size from 37% to 96% of the stance in the later part of the eccentric phase until shortly before takeoff. abd, knee abduction; ACLR, anterior cruciate ligament reconstruction; add, knee adduction; SLCMJ, single-legged countermovement jump; SPM, statistical parametric mapping.

The largest ESs were found in knee abduction moment (37-96% of stance; ES = -0.79) (Figure 2) and vertical CoM superior velocity (90-100% of stance; ES = −0.73) (Table 2, red shading).

### Double-Legged Drop Jump

**Figure 3.**
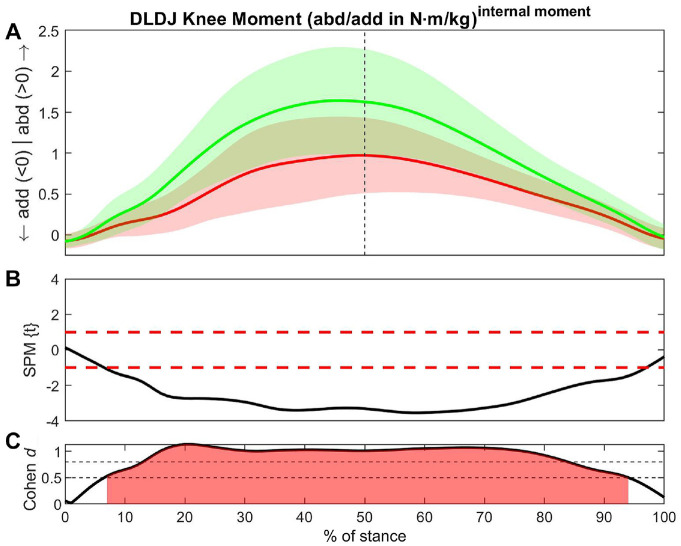
Differences in knee abduction moment between the ACLR and non-ACLR limbs during the double-legged drop jump. (A) Mean and SD clouds for the ACLR (red) and non-ACLR (green) limbs as a reference for movement. Knee adduction (negative values) and knee abduction (positive values) are centered around 0, with arrows pointing to the direction of movement. (B) The *t* statistic as a function of time describing the difference between the limbs. The curve exceeding the dashed red lines at >1 and <-1 (representing *P* < .05) indicated that a significant difference exists between the ACLR and non-ACLR limbs. (C) The effect size as a function of time describing the magnitude of the effect. The dotted black lines indicate the percentage of the stance with Cohen *d*≥ 0.5, with areas shaded in red indicating a strong effect size throughout that phase. The between-limb asymmetry was significantly different with a strong effect size from 7% to 94% of the stance in the earlier part of the eccentric phase until shortly before takeoff. abd, knee abduction; ACLR, anterior cruciate ligament reconstruction; add, knee adduction; DLDJ, double-legged drop jump; SPM, statistical parametric mapping.

The largest ESs were found in the knee abduction moment (7-94% of stance; ES = -0.95) (Figure 3) and ankle external rotation moment (11-87% of stance; ES = −0.92). Significant differences were also found in knee external rotation (1-96% of stance; ES = -0.77), vertical GRF (2-99% of stance; ES = −0.75), and ankle plantarflexion (4-32% of stance; ES = -0.72; and 67-95% of stance; ES = -0.71) (Table 2, red shading). Compared with the non-ACLR limb, the ACLR limb had larger values in the hip extension moment (53-61% of stance; ES = 0.55), in the contralateral CoM in the knee axis (23-43% of stance; ES = 0.55) and in the contralateral CoM in the ankle axis (84-100% of stance; ES = 0.54) (Table 2, green shading).

### Single-Legged Drop Jump

**Figure 4.**
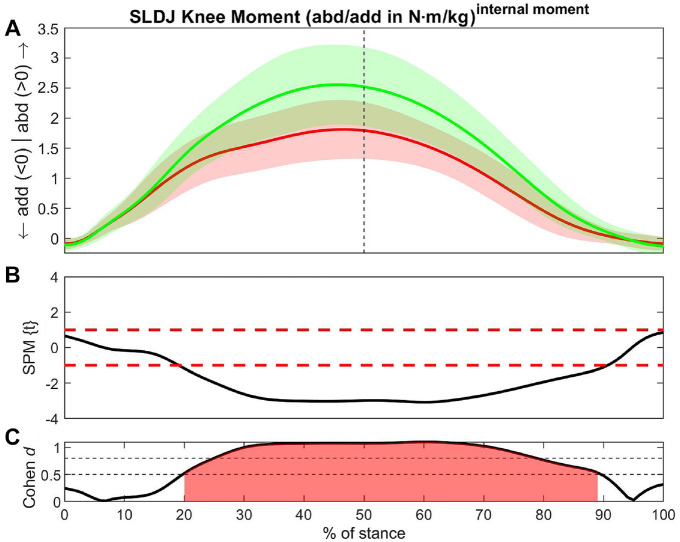
Differences in knee abduction moment between the ACLR and non-ACLR limbs during the single-legged drop jump. (A) Mean and SD clouds for the ACLR (red) and non-ACLR (green) limbs as a reference for movement. Knee adduction (negative values) and knee abduction (positive values) are centered around 0, with arrows pointing to the direction of movement. (B) The *t* statistic as a function of time describing the difference between the limbs. The curve exceeding the dashed red lines at >1 and <-1 (representing *P* < .05) indicated that a significant difference exists between the ACLR and non-ACLR limbs. (C) The effect size as a function of time describing the magnitude of the effect. The dotted black lines indicate the percentage of the stance with Cohen *d*≥ 0.5, with areas shaded in red indicating a strong effect size throughout that phase. The between-limb asymmetry was significantly different with a strong effect size between 20% and 89% of the stance in the midportion of the eccentric phase until the later part of the concentric phase. abd, knee abduction; ACLR, anterior cruciate ligament reconstruction; add, knee adduction; SLDJ, single-legged drop jump; SPM, statistical parametric mapping.

Large ESs were found in knee abduction moment (20% to 89% of stance; ES = -0.95) (Figure 4), vertical CoM velocity (48% to100% of stance; ES = −0.78), ankle external rotation moment (26% to82% of stance; ES = −0.77), and vertical GRF (49% to95% of stance; ES = −0.73) (Table 2, red shading). The posterior GRF showed a larger value in the ACLR limb versus non-ACLR limb at initial foot contact (4-19% of stance; ES = 0.54) but vice versa afterward (30% to38% of stance; ES = −0.58). Furthermore, the ACLR limb had larger values in hip extension moment (37% to46% of stance; ES = 0.56) (Table 2, green shading).

## Discussion

The findings of this study support our hypothesis that biomechanical and performance limb differences exist in female athletes across the kinetic chain during vertical jump testing at 9 months after ACLR. Limb asymmetries of >15% were detected for SL CMJ and DJ performance and quadriceps strength. All 4 vertical jump tests highlighted decreased vertical GRF, knee abduction moment, knee internal rotation angle, hip external rotation angle, ankle eversion, and external rotation moments in the ACLR limb. The greatest number of between-limb differences with large ESs was found for the DLDJ.

### LSI of Performance Variables

All results regarding performance differences between limbs were consistent with the literature. Limb asymmetries of >15% were found in maximum isokinetic quadriceps torque (LSI: 82.53%), as well as in jump height and RSI_mod_/RSI during the SLCMJ and the SLDJ (LSIs: SLCMJ 82.93%, 82.22%; SLDJ 79.27%, 76.31%). These results indicate incomplete rehabilitation.^8,9,22^ In contrast, completion and contact time of planned and unplanned CoD were not significantly different between limbs. These findings are consistent with the literature^15,16,27^ and indicate that CoD LSI has a poor ability to identify functional asymmetries compared with jump and biomechanical variables during rehabilitation after ACLR.^15^

### Biomechanical Differences Between Limbs

The difference between limbs in internal knee abduction moment demonstrated the strongest ES in all vertical jumps, especially during the SLDJ and DLDJ (ES = -0.95 for both). This finding supported the hypothesis that this deficit persists in female athletes 9 months after ACLR. Specifically, the limb that underwent ACLR consistently demonstrated lower internal abduction moments, pointing toward incomplete rehabilitation. Upon returning to play in an open environment, this characteristic may render the ACLR limb more susceptible to internal knee adduction moments. Consequently, this refers to knee abduction collapse, a well-known risk factor for ACL injury in females.^20^ Targeting this factor in rehabilitation is crucial. Frontal plane knee motions interact with proximal and distal joints in the kinetic chain,^30^ like the hip abduction angle, which was also decreased during the DLDJ. Furthermore, reduced hip abduction moments occurred with a large ES for the DLDJ (-0.70) and briefly for the SLDJ. King et al^16^ performed a comparable biomechanical analysis of SLDJ and DLDJ with males from the same cohort. They only showed differences of medium ES (–0.52 and –0.50) in hip abduction moments during the DLDJ. This difference between the sexes may be related to reduced hip control, which is a risk factor for ACL injury in females.^25^ Thus, it is particularly important that female athletes be examined for symmetry in hip biomechanics. If necessary, hip neuromuscular control exercises should be applied. Furthermore, vertical GRF shift to the contralateral limb can explain decreased hip abduction moments, coinciding with a more contralateral CoM position in the knee and ankle axis during the DLDJ. In addition, the ankle eversion moment is reduced in the ACLR limb during all 4 jumps and may influence passive external rotation at the knee,^3^ which can lead to ACL impingement.^20,33^

Appropriately, decreased knee internal rotation angles and ankle external rotation moments were observed for the ACLR limb during all jumping tests but with the largest ESs for the DLDJ (knee, –0.74; ankle, –0.92). Since greater knee external and internal rotation angles are associated with injury mechanisms,^33^ the risk for non-ACLR limb injury through strain may be increased.^20,33^ Correspondingly, a decreased knee external rotation moment was observed in the ACLR limb throughout a large part of the stance phase during the DLCMJ and DLDJ. The hip joint of the ACLR limb was less externally rotated during all jumps, which is associated with decreased knee abduction moment and, thus, increased ACL strain.^30^ Furthermore, the increased hip external rotator moment during the DLCMJ in the ACLR limb is related to hip extensor muscle performance. It should be targeted in rehabilitation to stabilize the frontal plane knee motion.^30^

Between-limb differences were found in vertical GRF, with large ESs for the DLCMJ (–0.71), DLDJ (–0.75), and SLDJ (–0.73) and a medium ES for the SLCMJ, as well as in CoM vertical velocity, with medium to large ESs for the SLCMJ (–0.59 at 37% to57% of stance and –0.73 at 90% to 100% of stance) and large ES for SLDJ (-0.78). Asymmetries during DL exercises are “learned nonuse” adaptations^1^ to shift functional demands to the healthy limb.^34^ Therefore, the findings indicate unloading force of the ACLR limb. In contrast, males did not show large ES for these variables during the DLDJ and SLDJ.^16^ Therefore, compensation patterns tend to appear predominantly in the frontal plane for females. Hence, it is important to assess and report females and males separately and target each more specifically to their sex-specific deficits. Apart from qualitative movement correction, rehabilitation should also include counterbalancing strength deficits since this is linked to jump height and RSI deficits,^14^ which again is connected to the decreased vertical GRF during the concentric phases.^17,34^

In addition to altered vertical GRF, decreased knee flexion angles and hip extension moments are related to the decreased quadriceps strength in the ACLR limb.^17,34^ Accordingly, knee flexion angles are reduced during SLCMJ and SLDJ as well as the knee extension moments primarily during DLDJ and SLDJ as well as briefly during DLCMJ. This contributes to an ACL injury mechanism in combination with knee abduction and external or internal rotation.^20,33^ Conversely, an increased hip extension moment was observed in the ACLR limb near eccentric-concentric transition during the DLDJ and SLDJ (DLDJ, 53% to61% of stance; SLDJ, 37% to46% of stance). Hip flexion improves energy absorption, decreases knee and ankle loads and contributes to vertical GRF alteration.^25^ Hewett et al^12^ speculated a better load distribution by musculature in a hinge body position with more extended knees and flexed hips. This also corresponds to a decreased posterior CoM position in the knee axis during SLDJ (ES = -0.59), which reduces knee extensor demand.^16^ However, the male counterparts showed a large ES (0.74) for this variable, which can be explained by reduced sagittal plane control in males.^13,14^ This suggests compensation patterns more on the sagittal plane compared with females. Despite the CoM position, utilization of fewer hip extensor muscles and more ankle plantarflexor muscles may explain this adjusted landing pattern, with decreased ankle plantarflexor moments during DLDJ (–0.72 at 4%to32% of stance; –0.71 at 67%to95% of stance) and SLDJ as well as reduced ankle dorsiflexion angles in SLCMJ and SLDJ.^5^

### Jump Test Choice Consideration

Exercises and variables need to be selected critically for each test purpose. DJ and isokinetic knee extension are superior to CoD for measuring readiness to return to play. Furthermore, the selection of an appropriate jump type is essential. Significant differences with a strong ES exist primarily in the concentric phase during CMJ and the eccentric and concentric phases during DJ. This may be explained by a drop from a height, including landing, and therefore more body deceleration during DJ compared with CMJ, resulting in more eccentric load on the body. Since landing is a major injury situation,^12^ DJ should be used over CMJ for return-to-play analysis.

The greatest number of biomechanical asymmetries is found in DLDJ, whereas the highest values of performance asymmetries are found in SLDJ. While SL jumps capture more of the jump ability, DL jumps quantify more kinetic compensatory strategies. Multiplanar and multijoint deficits highlight the importance of performance and biomechanical analysis.

The results also indicate the usefulness of waveform analysis, since discrete points such as peak values do not necessarily cover relevant phases, which were at different percentages of the stance for different exercises and variables. For example, during the DLDJ, the ankle plantarflexion moment peak value was between 2 detected phases, 4% to 32% (ES = −0.72) and 67% to 95% (ES = −0.71), whereas the external ankle rotation moment had a large ES (–0.92) between 11% and 87% of the stance. Analyzing discrete points of exercises was commonly used in past research but increases the risk of type I error.^28^

### Limitations

The study cohort consisted exclusively of injured female athletes; therefore, the results cannot be generalized to male athletes. Another limitation was needing a noninjured female control group to further strengthen the effects found, complicating further comparisons with male counterparts or an uninjured control group. In addition, only 1 time point was analyzed. Thus, no appropriate timeline for return to play could be concluded. Furthermore, 11 of the 67 participants had ACLR with a hamstring tendon graft, whereas the other 56 participants had a patellar tendon graft, which might affect the results. The athletes’ rehabilitation strategy was under the treating therapist's complete control. Hence, each athlete's rehabilitation process was individualized, potentially resulting in varying rehabilitation outcomes at the 9-month postoperative mark.

Internal joint moments and GRF were normalized to body mass, whereas the influence of height and limb length was neglected. More than 1/3 of the data had small phases without normal distribution. The alternative nonparametric test with 1000 permutations resulted in identical statements despite minimal smaller *P* values and larger ESs. For clarity, only parametric tests were reported. Given the exploratory nature of the research, including several jump tests and variables of the lower body during the entire stance phase might lead to overanalysis. However, this study design was chosen intentionally to explore all possible limb differences of females and classify the importance of exercises, kinematic and kinetic variables, and phases by ES. To minimize type I error, only ESs of ≥0.5 were considered.

## Conclusion

In the current study, female athletes demonstrated between-limb differences in jump performance and isokinetic strength as well as kinematics and kinetics, in particular the frontal and sagittal planes. The differences may indicate incomplete rehabilitation 9 months after ACLR and require more targeted interventions. Return-to-play testing in female athletes should examine the entire stance phase and focus, in addition to the knee abduction moment, as well as on contralateral compensation patterns identifiable with GRF or CoM shift, hip control deficits, knee flexion, and ankle moment alterations. Biomechanical asymmetries are greater during the DJ tests than CMJ, with the SL jump highlighting performance capacity and the DL jump focusing on kinetic and kinematic compensatory strategies.
